# Irradiance-Controlled Photoassisted Synthesis of Sub-Nanometre Sized Ruthenium Nanoparticles as Co-Catalyst for TiO_2_ in Photocatalytic Reactions

**DOI:** 10.3390/ma14174799

**Published:** 2021-08-24

**Authors:** Patricia García-Muñoz, Fernando Fresno, Javier Ivanez, Nicolas Keller

**Affiliations:** 1 Institut de Chimie et Procédés Pour l’Energie, l’Environnement et la Santé (ICPEES), CNRS/University of Strasbourg, 25 Rue Becquerel, 67087 Strasbourg, France; patricia.gmunoz@upm.es (P.G.-M.); ivanezcastellano@unistra.fr (J.I.); nkeller@unistra.fr (N.K.); 2 Department of Industrial Chemical and Environmental Engineering, Escuela Técnica Superior de Ingenieros Industriales (ETSII), Universidad Politécnica de Madrid, C/José Gutiérrez Abascal, 2, 28006 Madrid, Spain; 3 Photoactivated Processes Unit, IMDEA Energy, Avda. Ramón de la Sagra, 3, Móstoles, 28935 Madrid, Spain

**Keywords:** photoassisted synthesis, nanoparticles, TiO_2_, ruthenium, photocatalysis

## Abstract

Photoassisted synthesis is as a highly appealing green procedure for controlled decoration of semiconductor catalysts with co-catalyst nanoparticles, which can be carried out without the concourse of elevated temperatures, external chemical reducing agents or applied bias potential and in a simple slurry reactor. The aim of this study is to evaluate the control that such a photoassisted method can exert on the properties of ruthenium nanoparticles supported on TiO_2_ by means of the variation of the incident irradiance and hence of the photodeposition rate. For that purpose, different Ru/TiO_2_ systems with the same metal load have been prepared under varying irradiance and characterized by means of elemental analysis, transmission electron microscopy and X-ray photoelectron spectroscopy. The photocatalytic activity of the so-obtained materials has been evaluated by using the degradation of formic acid in water under UV-A light. Particles with size around or below one nanometer were obtained, depending on the irradiance employed in the synthesis, with narrow size distribution and homogeneous dispersion over the titania support. The relation between neutral and positive oxidation states of ruthenium could also be controlled by the variation of the irradiance. The obtained photocatalytic activities for formic acid oxidation were in all cases higher than that of undecorated titania, with the sample obtained with the lowest irradiation giving rise to the highest oxidation rate. According to the catalysts characterization, photocatalytic activity is influenced by both Ru size and Ru^0^/Ru^δ+^ ratio.

## 1. Introduction

Heterogeneous photocatalysis has gained renewed interest in the last years as a sustainable, solar-driven process with applications in the elimination of a plethora of hazardous substances in water and air, as well as in the production of solar fuels and chemicals by means of water splitting, photoreforming, CO_2_ reduction or nitrogen fixation [1,2]. In addition to the photocatalyst itself, the concourse of co-catalysts in heterogeneous photocatalysis, composed of metal or metal oxide nanoparticles deposited on the semiconductor photocatalyst, can significantly enhance critical aspects of the photocatalytic process such as light absorption and charge transfer; activation of reactant molecules; selectivity of the reaction and catalyst stability [3]. Photoassisted synthesis or photodeposition is based on the light-induced electrochemistry that occurs at the surface of a semiconductor upon irradiation. Thus, metal cations adsorbed on the semiconductor surface can be reduced (oxidized) by reactive electrons (holes) photo-generated by band-gap excitation, provided that the reduction potential of the conduction (valence) band is adequate. Among different alternatives, this is considered as a green and elegant preparation method for the obtainment of well-defined, small-sized supported nanoparticles with control over size, distribution and oxidation state [4]. In addition, it avoids the utilization of strongly reducing species by using only relatively harmless sacrificial agents such as alcohols, which can even be derived from biomass resources. It has been shown that, for example, the reductive photodeposition of Pt on TiO_2_, Ag on ZnO and Pt on CdS yields very attractive materials for photocatalytic applications [4]. Indeed, Pt is by far the metal to which photoassisted synthesis has been most widely applied because of its particular interest for water spitting and the very high photodeposition yield (usually stoichiometric) it achieves. Other metals, apart from the mentioned silver, include gold, palladium and rhodium [4]. Although the oxidative photoassisted synthesis of metal oxide nanoparticles is scarcer, the cases of PbO_2_ and RuO_2_ can be mentioned [5]. Ruthenium is actually a promising metal to be used as a (co)catalyst in both metallic and oxidized states not only in photocatalytic reduction and oxidation reactions, respectively [6,7], but also in other catalytic reactions such as those taking place in the conversion of biomass into fuels and value-added chemicals [8] or in electrochemical devices such as batteries [9], supercapacitors [10], electrolyzers [11] or fuel cells [12]. The photoassisted synthesis of ruthenium nanoparticles on semiconductor surfaces, however, has been considerably less studied than for other metals. As in the case of any metal and any catalytic application, control over size, distribution and oxidation state is highly desirable, and in this respect photodeposition appears as a highly appealing synthetic procedure, which can be carried out without the concourse of elevated temperatures, strong chemical reducing agents such as hydrogen or NaBH_4_ or applied bias potential and in a simple slurry reactor. In the particular case of Ru, it has been reported that the size of the nanoparticles can be controlled by varying the irradiation time [13], similarly to the case of photodeposited platinum [14,15].

The aim of this study is to evaluate the control that the photoassisted method can exert on the properties of ruthenium nanoparticles supported on TiO_2_ by means of the variation of the incident irradiance, which is a governing factor of any photoassisted reaction. For that purpose, different Ru/TiO_2_ systems with the same metal load have been prepared under varying irradiance and characterized by means of ICP-OES analysis, TEM and XPS. The activity of the so-obtained materials has been evaluated using the photocatalytic oxidation (PCO) of formic acid in water under UV-A, which is well suited as a model reaction for several reasons: On the one hand, its oxidation via hole capture directly proceeds to carbon dioxide [16,17], thereby eliminating the possible effect of reaction intermediates and facilitating kinetic analyses; on the other hand, it only absorbs light in the UV-C region, where the lamps commonly used in photocatalytic reactions show no emission, so that its absorption does not play any role in the reaction; furthermore, it is per se a pollutant of interest due to its appearance as the last organic intermediate in the degradation of manifold refractory water pollutants [18,19,20,21,22].

## 2. Materials and Methods

### 2.1. Synthesis of Photocatalysts

Aeroxide^®^ P25 TiO_2_ from Evonik Industries (Essen, Germany) was used (anatase + rutile 80:20, BET 50 m^2^g^−1^) as semiconductor support for Ru photodeposition. In a typical procedure, RuCl_3_·xH_2_O (40% Ru min. content, Sigma-Aldrich), in the amount for nominal 0.5 wt.% Ru, was dissolved in 10 mL methanol and then diluted with 90 mL ultrapure water. TiO_2_ (100 mg) was then suspended in this solution and maintained with vigorous magnetic stirring in the dark for 1 h in order to ensure the attainment of the adsorption–desorption equilibrium. Irradiation was provided by a box equipped with five independent UV lamps (λ_max_ = 365 nm, Philips 24 W/10/4P), while the temperature was kept constant at 20 °C through a double-walled jacket fed by thermostated flowing water. Experiments with different irradiance controlled by the number of lamps were carried out, corresponding to 14.3, 27.8, 41.2 and 69.6 Wm^−2^ as determined experimentally with a spectroradiometer (RPS900-W International Light Technology, Peabody, MA, USA) by considering the attenuation by the reactor walls. The so-obtained Ru/TiO_2_ samples are hereafter referred to as Ru-14, Ru-28, Ru-41 and Ru-70, respectively. At regular intervals, 0.5 mL of the reaction medium was sampled and filtered through a 0.20 µm porosity filter (Aireka Cells, Hong Kong) in order to remove the TiO_2_ powder, and the ruthenium concentration in solution was followed with a UV-vis spectrophotometer (VWR UV-1600 PC, Radnor, PA, USA) by monitoring the evolution of the main absorption band at 325 nm of the Ru precursor.

### 2.2. Characterization Techniques

The Ru content in the catalysts was determined by inductively coupled plasma optical emission spectroscopy (ICP-OES) carried out on an Optima 7000 DV spectrometer (Perkin Elmer, Waltham, MA, USA) after microwave-digestion of the solid samples in a HNO3:HCl:HF 3:2:3 mixture. Transmission electron microscopy (TEM) was performed by using a JEOL 2100F microscope (Tokyo, Japan) with a point resolution of 0.2 nm working at 200 kV, after depositing the powdered samples on holey carbon membrane coated copper grids from ultrasound-irradiated ethanolic suspensions. X-ray photoelectron spectroscopy (XPS) was performed on a ThermoVG Multilab ESCA3000 spectrometer (Thermo Fisher Scientific, Waltham, MA, USA) equipped with an Al Kα anode (hν = 1486.6 eV). The energy scale was adjusted using the adventitious carbon C 1s band at 284.6 eV. Contributions with Doniach–Sunjic shape [23] and Shirley-type background [24] were used. Ru/Ti surface atomic ratios were derived from the peak areas in the Ru 3d and Ti 2p high-resolution spectra by using the appropriate cross-sections-based sensitivity factors as determined by Scoffield [25].

### 2.3. Photocatalytic Reactions

The photocatalytic degradation of formic acid was carried out at 20 °C in the same box as the photodeposition with two parallel lamps (i.e., 27.8 W m^−2^). In a typical procedure, 40 mg of photocatalyst was suspended in 40 mL of 100 ppm aqueous HCOOH (Sigma Aldrich, St. Louis, MO, USA, >95%) solution and remained under stirring for 30 min until the adsorption–desorption equilibrium was reached. Afterwards, the lamps were switched on, and the formic acid concentration decay was followed by monitoring the absorption band at 210 nm by using a UV-vis spectrophotometer (VWR UV-1600 PC). In order to confirm the viability of this analysis technique, total organic carbon (TOC) analyses were also carried out by using a Shimadzu TOC-L analyzer (Kyoto, Japan).

## 3. Results and Discussion

### 3.1. Synthesis and Characterization of TiO_2_-Supported Subnanometre Ru Particles

**Figure 1:**
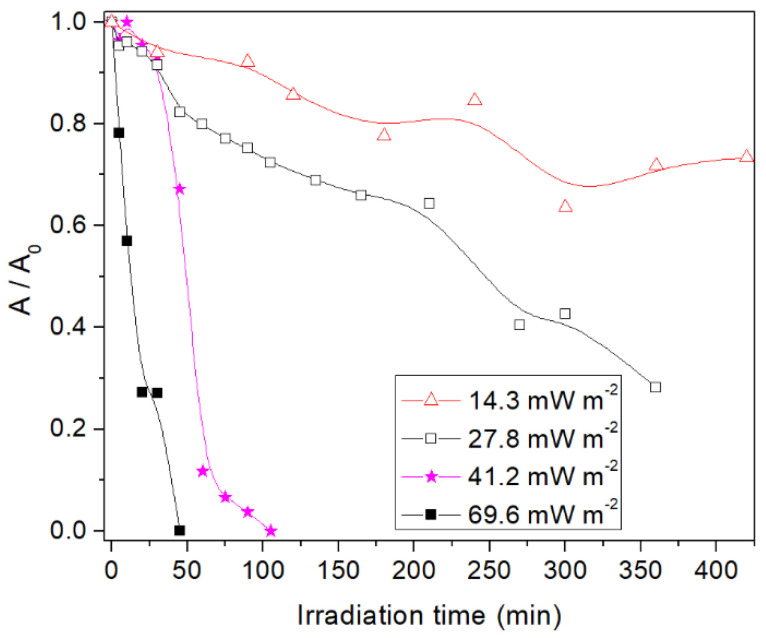
Decay of Ru species in solution monitored by the absorbance at 325 nm during photodeposition on TiO_2_ with different incident irradiances. Solid lines are guides to the eye only.

The evolution of ruthenium species in solution during the photoassisted synthesis on the TiO_2_ support, as measured by UV-vis spectroscopy, shows different decay rates with different incident irradiance (Figure 1). Experiments in the dark or in the absence of TiO_2_ gave rise to stable Ru concentrations [26], in the first case after the establishment of the adsorption/desorption equilibrium at the titania surface. As a general trend, a faster decay of dissolved Ru species absorbing at 325 nm with higher incident irradiance is observed, indicating the effect of photon flux on reaction kinetics. Induction periods appear at the beginning of the reactions, after which the reaction rates increase. This could be related with a multi-step reduction through the different oxidation states of ruthenium. With increasing irradiance, these periods tend to be shorter, which could be also related to faster reduction kinetics and therefore a faster evolution of the resulting solid.

The characteristics of the so-obtained Ru/TiO_2_ materials are collected in Table 1. The yield of ruthenium photodeposition is similar in all cases and in good accordance with previous results using RuCl_3_ as precursor [7]. The apparent contradiction between the similar Ru contents and the different absorbance at 325 nm after each synthesis reveals that some Ru species remain in the solution, even if not detected by UV-vis spectroscopy in the used wavelength interval, and that the disappearance of the Ru(III) species absorbing at 325 nm does not directly mean that Ru is deposited as a solid on the surface of titania, further suggesting that the reduction of Ru(III) is only one of several steps in the photodeposition process, as mentioned in the previous paragraph.

**Figure 2:**
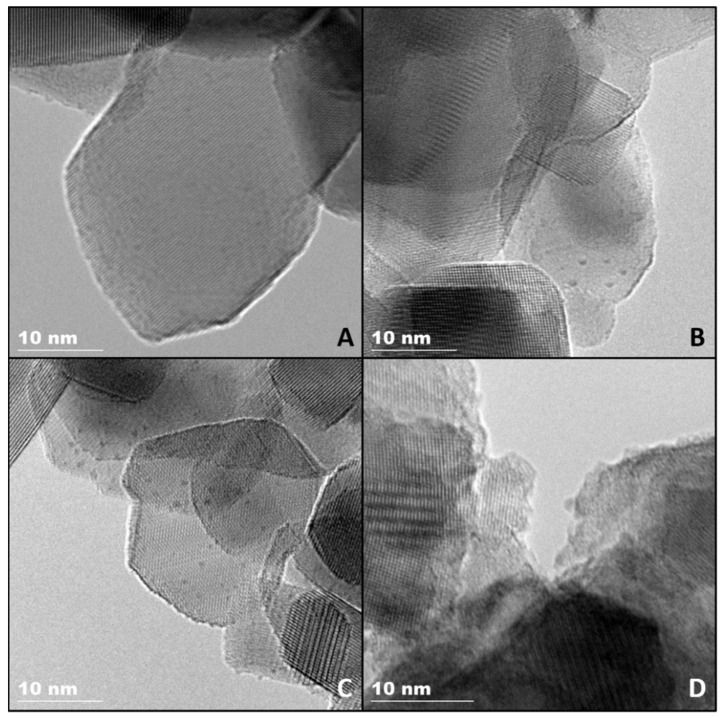
Example TEM images of Ru-14 (**A**), Ru-28 (**B**), Ru-41 (**C**) and Ru-70 (**D**).

**Figure 3:**
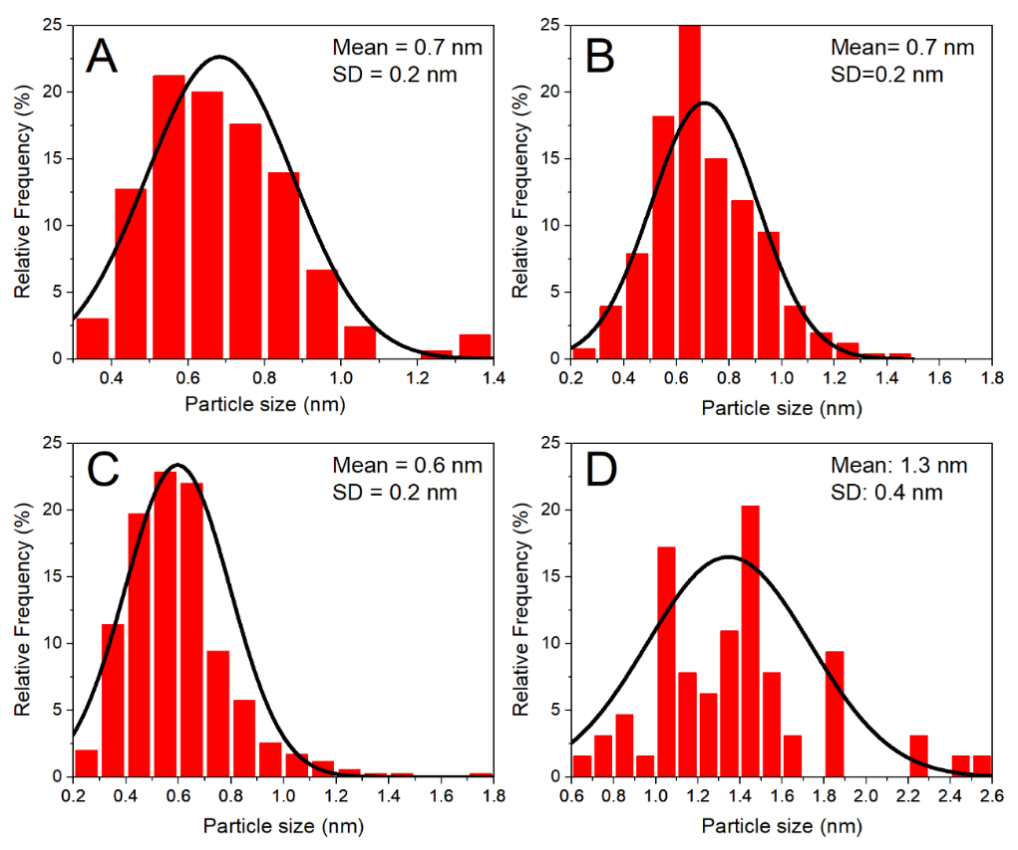
Ru particle size distribution histograms from TEM images of Ru-14 (**A**), Ru-28 (**B**), Ru-41 (**C**) and Ru-70 (**D**).

TEM images (Figure 2) were used to analyze the particle size of the photodeposited Ru particles, and the obtained mean sizes and distribution widths are included in Table 1 while histograms are shown in Figure 3. Fairly narrow distributions are obtained in all cases, with particles near or below one nanometer on average. An effect of irradiance on the particle size, however, is only evident in the sample obtained with the highest irradiance that is characterized by a slightly lower dispersion in comparison to the rest of samples that shows similar size distributions with sub-nanometre mean sizes and very high dispersions.

**Figure 4:**
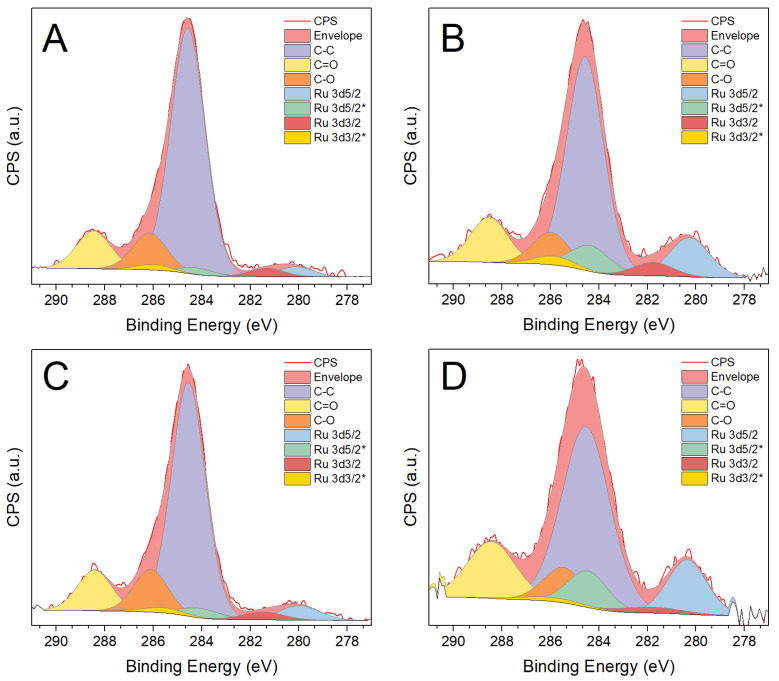
X-ray photoelectron spectra of Ru/TiO_2_ catalysts in the Ru 3d + C 1s region: Ru-14 (**A**), Ru-28 (**B**), Ru-41 (**C**) and Ru-70 (**D**). Asterisks denote the Ru^δ+^ components.

X-ray photoelectron spectra (Ru 3d + C 1s region in Figure 4) reveal similar Ru/Ti surface atomic ratios for all samples (included in Table 1). The apparent contradiction with the larger mean particle size and the slightly lower associated dispersion observed for the sample Ru-70 reflects the fact that Ru particle sizes remain all within the analysis depth of the technique. The influence of photodeposition irradiance on the oxidation state of ruthenium, however, is more evident. Thus, higher irradiance appears to produce ruthenium nanoparticles with a higher metallic state content. The difference is significant even for samples with similar particle size, revealing an effect beyond that caused by the surface-to-volume ratio. In this respect, considering that the possible formation of a Schottky barrier may favour electron transfer from TiO_2_ to ruthenium, more conduction band electrons due to higher irradiance would imply that the reduction of Ru^3+^ proceeds to Ru^0^ to a larger extent. In turn, Ru^δ+^ species may arise either from a natural oxidation of the Ru surface or from the existence of Ti-O-Ru interfacial species that have been shown to exist also with other TiO_2_-supported metals such as silver [27]. A co-existence of both mechanisms could imply that, for the smallest particles, most of the Ru atoms might actually be in a positive oxidation state, and those particles may, therefore, be considered as RuO_x_.

### 3.2. Influence of Ru Nanoparticle Characteristics on Photocatalytic Activity

**Figure 5:**
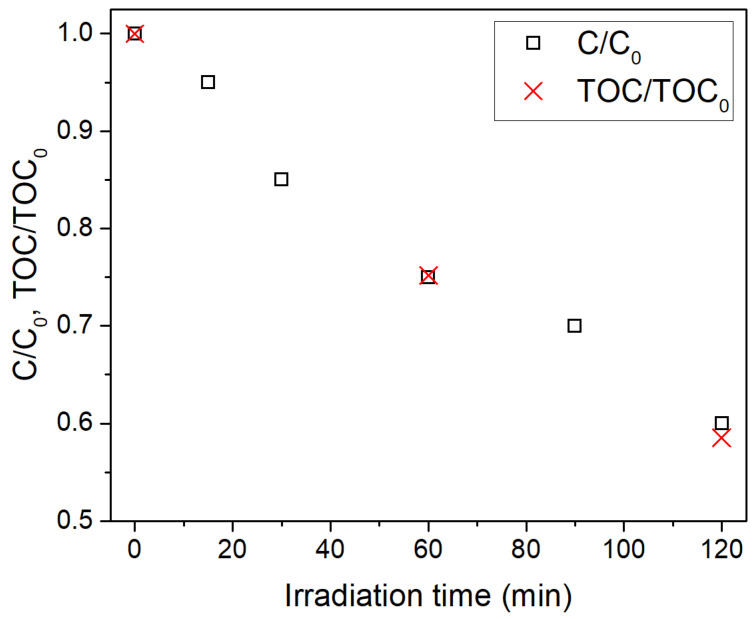
Comparison of photocatalytic degradation of formic acid over TiO_2_-P25 monitored by UV-vis spectra (C/C_0_) and total organic carbon measurements (TOC/TOC_0_).

The photocatalytic activity of the different Ru/TiO_2_ samples, together with that of the bare TiO_2_ P25 support, was analyzed for the degradation of formic acid followed by UV-vis absorption spectroscopy. The accuracy of this analytical procedure was checked by additionally following the reaction by TOC using TiO_2_ P25 as a catalyst. As shown in Figure 5, there is a perfect correlation between the two analytical techniques with an error smaller than 3% at the end of the analyzed reaction period, demonstrating that HCOOH degradation proceeds directly to CO_2_.

**Figure 6:**
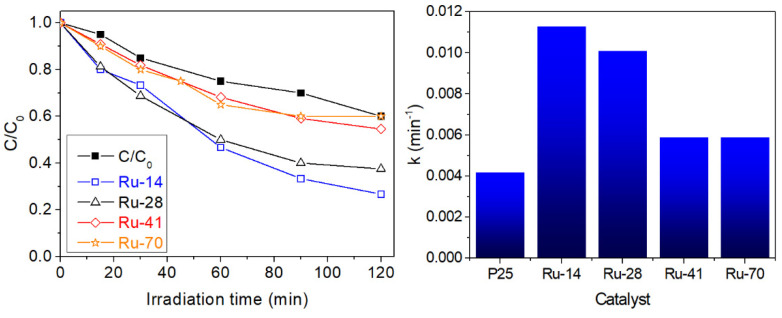
Formic acid oxidation with Ru/TiO_2_ catalysts and TiO_2_-P25 as reference. Left panel: concentration decay with irradiation time. Right panel: apparent first-order kinetic constants fitted from semi-logarithmic plots.

Applying photo-synthesized Ru/TiO_2_ materials to this reaction gave rise to the degradation curves vs. irradiation time shown in Figure 6, where the reaction performed with the unmodified P25 titania has been added for comparison. The data are well fitted by a first-order reaction rate following a Langmuir–Hinshelwood model for low substrate concentrations, as expected in a photocatalytic process [28]. The so-obtained rate constants are included in Figure 6. First of all, it is observed that all Ru-decorated samples overperform the activity reached by the bare P25 titania, highlighting the role of Ru nanoparticles as co-catalyst for PCO reactions. Furthermore, the degradation kinetics on the different Ru/TiO_2_ samples reveal interesting differences. Although the trend is not monotonic, there is a substantial effect on the activity of the FA oxidation depending on the photoassisted synthesis irradiance. Thus, the catalysts prepared at lower irradiances reach higher mineralization rates. As deduced from the data in Table 1, this can be related either with variations in particle size, if comparing the two extreme situations Ru-14 and Ru-70, or with modifications in the oxidation state as revealed by X-ray photoelectron spectra. In the former case, the positive effect of the small particle size of co-catalysts is well established in photocatalysis, providing a higher number of active sites per unit area, higher reactivity due to the higher presence of undercoordinated sites or even different electronics due to quantum size effects [29]. On the other hand, the differences observed between the samples with similar particle size suggest a significant influence of the latter parameter, which is the sample with the highest ratio of Ru^δ+^ species giving rise to the highest activity. In this respect, as it was stressed in the introduction, RuO_x_ particles are generally found as good oxidation catalysts, while rather metallic species tend to promote reduction reactions [7,30], due to their different band alignment with respect to TiO_2_ [31,32]. Further investigations are underway in order to discriminate between those effects, as well as to study the extension of this research to reduction-intensive reactions (e.g., photocatalytic hydrogen evolution or photocatalytic CO_2_ reduction) in order to explore the implications of the control of size and oxidation state of the nanoparticles in the optimisation of oxidation/reduction reactions.

## 4. Conclusions

Subnanometre-sized Ru nanoparticles were successfully synthesized on TiO_2_ by using a photoassisted method based on photodeposition. Particles with sizes around or below one nanometer were obtained, depending on the irradiance employed in the synthesis, with narrow size distribution and good dispersion over the titania support. The relation between neutral and positive oxidation states of ruthenium could also be controlled by the variation of the irradiance. The obtained photocatalytic activities for formic acid oxidation were in all cases higher than that of undecorated titania, with the sample obtained with the lowest irradiation giving rise to the highest reaction rate. According to the catalysts characterization, photocatalytic activity is influenced by both the Ru size and Ru^0^/Ru^δ+^ ratio. Although further work is needed to attain fine control over the properties of the materials through irradiance and to explore the different applications of this control, the present work opens up interesting implications in the search for green, highly controllable material synthesis methods for environmental catalysis and related applications.

## Figures and Tables

**Table 1:** Physicochemical characteristics of Ru/TiO_2_ materials obtained by photoassisted synthesis.

Sample	Synthesis Time (h)	Ru wt.% ^a^	Photodeposition Yield (%) ^b^	Ru/Ti at. ^c^	Ru^0^/(Ru^0^ + Ru^δ+^) ^c^	Ru Mean Size (nm) ^d^
Ru-14	7.5	0.21	42	0.013	0.48	0.7 ± 0.2
Ru-28	6	0.23	46	0.014	0.66	0.7 ± 0.2
Ru-41	2	0.20	40	0.012	0.65	0.6 ± 0.2
Ru-70	1	0.21	42	0.011	0.85	1.3 ± 0.4

^a^ From ICP-OES. ^b^ Nominal wt.% is 0.5 in all cases. ^c^ From XPS. ^d^ From TEM.
